# Concordance of Clinical, Histologic and Direct Immunofluorescence Findings in Patients with Autoimmune Bullous Dermatoses in Vietnam

**DOI:** 10.3390/dermatopathology10010004

**Published:** 2023-01-10

**Authors:** Giang Huong Tran, Nhan Thi Ai Le, Minh Hoang Dang, Thao Thi Phuong Doan, Thuy L. Phung

**Affiliations:** 1 Department of Pathology, University of Medicine and Pharmacy at Ho Chi Minh City, Ho Chi Minh City, Vietnam; 2 Department of Pathology, College of Medicine, University of South Alabama, Mobile, AL 36617, USA

**Keywords:** autoimmune bullous dermatoses, direct immunofluorescence stains, subepidermal bullous disorders, intraepidermal bullous disorders

## Abstract

*Introduction*: Autoimmune bullous dermatoses (ABD) represent a heterogeneous group of blistering disorders that may be debilitating with high morbidity. Clinical, histological, and direct immunofluorescence (DIF) studies are essential in establishing an accurate diagnosis of ABD, which is essential for its clinical management. Our study objective was to perform a systematic evaluation of ABD cases in a patient population at an academic medical center in Ho Chi Minh City, Vietnam, and determine the degree of concordance of clinical, histological, and DIF findings in ABD. *Methodology*: A systematic retrospective cross-sectional study was performed on 92 patients diagnosed with ABD by clinical, histological, and DIF studies at the University of Medicine and Pharmacy in Ho Chi Minh City, Vietnam, between September 2019 and September 2021. The clinical histories, H and E stained tissue sections, and DIF stains were evaluated by pathologists at the University of Medicine and Pharmacy. *Results*: ABD was evaluated as a whole and subdivided into an intraepidermal blister subgroup and a subepidermal blister subgroup. The analysis of paired diagnostic methods (clinical, histological, and DIF) for concordance with the final diagnosis was performed and showed that there were no statistically significant differences between the paired methods (McNemar’s test, *p* > 0.05). There was moderate concordance between the clinical, histological, and DIF diagnoses among all ABD cases (Brennan-Prediger coefficient Kappa test, κ_BP_ = 0.522, CI = 0.95). In the intraepidermal blister subgroup, the diagnostic accuracies of the histology and DIF stains were comparable to each other, and both were more accurate than a clinical diagnosis alone. In the subepidermal blister subgroup, there was no statistically significant difference in each pair of the three diagnostic methods (clinical, histological, and DIF) (McNemar’s test, *p* > 0.05). The concordance between the clinical, histological, and DIF diagnoses was high for the intraepidermal blister subgroup (Kappa test, κ_BP_ = 0.758, CI = 0.95). However, the concordance between the clinical, histological, and DIF diagnoses was slight for the subepidermal blister subgroup (Kappa test, κ_BP_ = 0.171, CI = 0.95). *Conclusion*: Histological evaluation is highly accurate in the diagnosis of the intraepidermal blister subgroup, but it is not as accurate in the diagnosis of the subepidermal blister subgroup in the Vietnamese patient cohort in which clinical, histological, and DIF studies were performed. DIF stains are a crucial diagnostic tool for ABD in this patient population.

## 1. Introduction

Autoimmune bullous dermatoses (ABD) represent a heterogeneous group of rare blistering disorders characterized by antibody-mediated autoimmune responses against the structural components of the skin and mucosa, resulting in blistering eruptions [1,2,3]. Bullous disorders are categorized into subepidermal and intraepidermal blister subgroups according to where in the epidermis the split occurs. Subepidermal bullous disorders include bullous pemphigoid (BP), mucous membrane pemphigoid (MMP), pemphigoid gestationis (PG), linear IgA dermatosis (LAD), anti-p200/laminin γ1 pemphigoid (p200 pemphigoid), lichen planus pemphigoid (LPP), bullous systemic lupus erythematosus (BSLE), epidermolysis bullosa acquisita (EBA), and dermatitis herpetiformis (DH). Intraepidermal bullous disorders include pemphigus vulgaris (PV), pemphigus vegetans (PVe), pemphigus foliaceus (PF), pemphigus erythematosus (PE), pemphigus herpetiformis (PH), endemic pemphigus foliaceus/fogo selvagem (EPF), IgA pemphigus (IAP), and paraneoplastic pemphigus (PNP) [1,2,3]. The incidence of bullous disorders is generally low and variable worldwide [4,5,6,7]. These disorders have serious morbidities and could even be fatal, and therefore would require early diagnoses and therapeutic interventions [8,9]. The histopathologic evaluation of skin biopsies for autoimmune bullous disorders is important for accurate diagnosis. Direct immunofluorescence (DIF) microscopy provides a highly valuable tool to determine the characteristics of bullous disorders [10,11,12]. There is very limited information on the clinical, histological, and DIF correlation in the patient population with ABD in Vietnam. Our aim was to determine the correlation among these three diagnostic modalities in patients in Vietnam with a clinical diagnosis of ABD.

## 2. Methodology

A cross-sectional retrospective chart review study was conducted on 92 cases diagnosed with ABD and DIF stains in the Department of Pathology, University of Medicine and Pharmacy in Ho Chi Minh City, Vietnam, from September 2019 to September 2021. The clinical histories and histopathology work-ups were retrospectively collected from patients’ medical records. The inclusion criteria of this study were:

(a) Adequate clinical history and description of lesions;
(b) Skin and/or mucosal biopsies were sent to the pathology laboratory of the University of Medicine and Pharmacy at Ho Chi Minh City with proper preservation and transportation for the DIF assays;
(c) Hematoxylin and eosin (H and E) stained slides of the skin biopsies were available for evaluation;
(d) DIF-stained slides of the skin biopsies were available for evaluation;
(e) Cases with a missing epidermis on the DIF and/or H and E slides were excluded from the study. Also excluded were cases with negative DIF results, including cases with prior treatments, and therefore may have had negative DIF results.

The clinical data were collected from patients’ medical records. For each patient, two biopsy specimens were obtained and processed: one biopsy for the routine H and E stains and the other biopsy for the DIF stains. A shave biopsy specimen was obtained from the edge of an intact blister consisting of the edge of a bulla and adjacent non-bullous “normal” skin. The specimen was fixed in 10% neutral-buffered formalin and then processed in paraffin as per standard histological procedures. Four micron-thick tissue sections were cut for hematoxylin and eosin (H and E) staining. Another shave biopsy specimen was taken from non-bullous erythematous skin within 1 cm of a bulla for the DIF stains. This specimen was placed on saline-soaked gauze and transported to the laboratory within 24 h. The specimen was embedded in the Optimal Cutting Temperature (OCT) compound and frozen at −20 °C in a cryostat. Six 4 µm-thick tissue sections of the specimen were cut onto charged glass slides and air-dried for 15 min. The slides were fixed in acetone at −20 °C for 10 min, then air dried for five minutes, and permeabilized with 0.3% Triton X-100/phosphate-buffered saline (PBS) for 10 min before washing in PBS. The slides were then incubated with a fluorescein isothiocyanate (FITC)-labeled primary antibody to IgA, IgG, IgM, C3, and fibrinogen at 37 °C for one hour, then rinsed three times in PBS for five minutes each rinse to remove the unbound antibodies. A negative control slide was prepared in the same manner as the specimen slides without the addition of an antibody. All slides were mounted with buffered glycerin. The DIF-stained slides were examined by a pathologist to determine the split location (subepidermal or intraepidermal) and pattern (linear or granular) of the immunoglobulin deposition.

The histological diagnoses were based on the presence of classic microscopic features. The microscopic assessment of the H and E slides included the level of split formation (superficial, suprabasal, subepidermal, mixed, or other levels), the presence of acantholysis, and the predominant inflammatory cells (e.g., neutrophils, eosinophils, or mononuclear cells) [1,2,3] (Table 1). For example, the histological diagnosis of “pemphigus foliaceus” was based on the presence of a blister or acantholysis occurring in the subcorneal or granular layer in the epidermis with fibrin and some neutrophils. The DIF findings showed intercellular staining for IgG and C3 in the upper layer of the epidermis, sparing the basal layer. The histological diagnosis of “pemphigus vulgaris” was based on the presence of suprabasal bullae with acantholysis that may have extended down the adnexal structures. Acantholysis of keratinocytes often showed a “tombstone” appearance. The bulla cavity often contained acantholytic cells, some eosinophils, and neutrophils. The DIF findings showed the deposition of IgG (and sometimes C3, IgM, and IgA) in the intercellular regions of the epidermis with a “fishnet” pattern. The histological diagnosis of subepidermal bullous disorders, especially “bullous pemphigoid,” was based on the presence of unilocular subepidermal blisters with eosinophils, neutrophils, and lymphocytes in the blister and the dermis. Bulla taken in normal-appearing skin often showed a sparse dermal inflammatory infiltrate, whereas bulla from lesions with an erythematous base often had more dense inflammatory infiltrates in the dermis. The DIF findings showed a linear and homogeneous deposition of IgG and C3 along the basement membrane zone.

The DIF diagnoses were based on typical immunoglobulin depositions. The DIF slides were examined to determine the primary sites of immune deposition, classes of immunoglobulin deposition (IgG, IgA, IgM, C3, and fibrinogen), stain intensity, and patterns of immunoglobulin deposition [10,11,12,13,14,15] (Table 2). The staining patterns were classified into five subgroups: epidermal cell pattern, linear basement membrane zone (BMZ) pattern, granular BMZ pattern, shaggy BMZ pattern, vascular, and other patterns. In cases demonstrating more than one type of immunoglobulin deposition, the deposition with the highest intensity was recorded. In cases demonstrating a linear BMZ pattern, the user-rated or n-serrated pattern was identified. The final diagnoses were established based on a combination of the clinical, histological, and DIF findings.

Statistical methods: The data analysis was performed using Microsoft Excel and Software for Statistics and Data Science (Stata) version 14.0. The differences between clinical and histopathologic diagnoses, between clinical and DIF diagnoses, and between histopathology and DIF diagnoses were determined using McNemar’s test, which is used to determine if there are differences regarding a dichotomous dependent variable between two related groups. The overall agreement between the clinical, histopathological, and DIF diagnoses was assessed using the Brennan-Prediger coefficient using Kappa statistics. The statistical significance was set at a 95% confidence interval (CI = 95%).

## 3. Results

### 3.1. Spectrum of Autoimmune Bullous Dermatoses

We evaluated a total of 92 cases of ABD from 2019 to 2021, consisting of 55 cases of intraepidermal blisters and 37 cases of subepidermal blisters. The microscopic assessment of the H and E slides included the level of split formation (superficial, suprabasal, subepidermal, mixed, or other levels), the presence of acantholysis, and the predominant inflammatory cells (e.g., neutrophils, eosinophils, or mononuclear cells) [1,2,3] (Table 1 in Section 2 Methodology). The majority of ABD cases was pemphigus vulgaris (44.6%), followed by bullous pemphigoid (29.4%), pemphigus foliaceus (14.1%), linear-IgA dermatosis (8.7%), and single cases of IgA pemphigus, bullous systemic lupus erythematosus, and dermatitis herpetiformis. The patient characteristics and prevalence of ABD encountered in the study are summarized in Table 3. 

The patients with intraepidermal blisters were between 11 and 82 years of age, with a mean age of 52 years. There were two pediatric cases of intraepidermal blisters: an 11-year-old female with PV and an 11-year-old male with IAP. There was greater than twice the number of females affected than males.

The patients with subepidermal blisters were between 10 and 95 years of age, with a mean age of 60 years. There was one pediatric case of subepidermal blisters: a 10-year-old male with BP, a 14-year-old male with LAD, and a 16-year-old female with BSLE. There was an equal ratio of females and males affected. All LAD cases had an onset after puberty, with 3/8 (37.5%) patients younger than 19 years old. 

### 3.2. Concordance between the Clinical, Histopathological, and Direct Immunofluorescence Stains in ABD

We evaluated the extent of concordance between paired diagnostic methods using McNemar’s test to determine if differences existed regarding a dichotomous dependent variable between two paired or related groups. The paired diagnostic methods included clinical diagnosis and histological diagnosis, clinical diagnosis and DIF diagnosis, and DIF diagnosis and histological diagnosis. A result is designated as positive (+) if it is concordant with the final diagnosis. A result is designated as negative (−) if it is discordant with the final diagnosis. The McNemar statistics contingency table comparing the efficacy of the paired diagnostic methods for the intraepidermal bulla and subepidermal bulla subgroups is shown in Table 4.

In a total of 92 ABD cases, the differences between the clinical diagnoses and histological diagnoses, between the clinical and DIF diagnoses, and between the histological and DIF diagnoses were not statistically significant (mid-*p*-McNemar’s test, α = 0.05) (Table 5). The agreement between the clinical, histological, and DIF diagnoses was statistically significant, with the Brennan-Prediger coefficient (κ_BP_) of 0.522 (CI = 0.95), which indicates a moderate level of agreement.

Within the intraepidermal blister subgroup, the differences between the clinical diagnosis and histological diagnosis (McNemar’s test mid-*p*-value = 0.021) and between the clinical and DIF diagnoses (McNemar’s test mid-*p*-value = 0.008) were statistically significant. The histological examination and DIF staining provided comparable results (McNemar’s test with mid-*p*-value = 1.000) (Table 6). The concordance between the clinical, histological, and DIF diagnoses was statistically significant, with a Brennan-Prediger coefficient κ_BP_ coefficient of 0.758 (CI = 0.95), indicating a substantial level of agreement.

Within the subepidermal blister subgroup, the in-pair differences of the three pairs of diagnostic methods were not statistically significant according to the mid-*p*-McNemar’s test (Table 7). The concordance between the clinical, histological, and DIF diagnoses was statistically significant with a κ_BP_ of 0.171 (CI = 0.95), which indicates a slight extent of agreement.

There were discordant findings in the clinical/DIF pair of diagnostic methods for subepidermal blisters. Examples of such discordant cases are shown in Table 8 and include cases in which the clinical diagnoses were linear IgA dermatosis, herpes-associated erythema multiforme, dermatitis herpetiformis, and pemphigus vulgaris, while the DIF diagnosis was bullous pemphigoid, and the final diagnosis was bullous pemphigoid.

## 4. Discussion

In the Vietnamese patient cohort of this study, pemphigus vulgaris and bullous pemphigoid were the two most prevalent disorders of ABD, with pemphigus vulgaris accounting for 74.5% of the intraepidermal blister subgroup and bullous pemphigoid accounting for 73.0% of the subepidermal blister subgroup. Pemphigus vulgaris was more common than bullous pemphigoid (41 cases of pemphigus vulgaris versus 27 cases of bullous pemphigoid). The findings appear to be comparable with those reported in the literature [7]. In our study, pemphigus foliaceus accounted for 23.6% of the intraepidermal blister subgroup, which is higher than those reported in Turkish and Iranian populations [16,17,18]. The prevalence of linear IgA dermatosis (8.7%) in our patient population is significantly higher than those in the populations in Singapore (3%), France (5.3%), and Germany (5%) [4,6,19]. The only patient in our study with dermatitis herpetiformis had a Caucasian heritage, and this correlates with a higher incidence of dermatitis herpetiformis in the European population and much rarer in the African and Asian populations [20].

This study shows a marked predilection for females in the intraepidermal blister subgroup with female-to-male ratios of 2.7:1 for pemphigus vulgaris, and 2.2:1 for pemphigus foliaceus. A female predilection for intraepidermal blisters was also reported in other studies in different populations and geographic regions [7]. The predilection for females was also reported in the subepidermal blister subgroup, with female-to-male ratios ranging from 1:1 to 5:1 [21]. However, our study did not show a gender predilection for subepidermal blisters, but this may be due to the small size of the study cohort.

We observed that most patients in the intraepidermal blister subgroup were 30 to 59 years of age, with an average age of 52 years, which is comparable with data reported in the literature [21]. The percentage of patients with bullous pemphigoids over 60 years was 63.0%, which is significantly lower than that reported in the United Kingdom population (87.9%) and the US population (93.1%) [22,23], and this could be related to the overall younger age of the Vietnamese population as compared with the UK and US populations.

We observed that the agreement between the clinical, histological, and DIF diagnoses was statistically significant, with a Brennan-Prediger coefficient (κ_BP_) of 0.522 (CI = 0.95), which indicates a moderate level of agreement. There was no statistically significant difference between the clinical and histological diagnoses, between the clinical and DIF diagnoses, and between the histological and DIF diagnoses (mid-*p*-McNemar’s test, *p* > 0.05). There were three cases in which the initial clinical diagnosis differed significantly from the final diagnosis. There was a case that had an initial clinical diagnosis of allergic contact dermatitis, which was, in fact, pemphigus foliaceus; a case with a clinical diagnosis of allergic contact dermatitis that turned out to be pemphigus vulgaris; and a case with clinical diagnosis of herpes-associated erythema multiforme that turned out to be bullous pemphigoid. DIF stains performed on these three cases showed immunoglobulin deposition characteristics of immune bullous disorders.

We encountered cases of pemphigus vulgaris that were histologically inconclusive due to the lack of vesiculobullous appearances in the biopsies of the lesions. In these cases, the DIF stains of the biopsies of perilesional skin showed a typical “fishnet” pattern of IgG deposition in the intercellular regions in the epidermis, consistent with pemphigus vulgaris. DIF stains play a key role in the final diagnosis of ABD. In cases with IgG and C3 depositions along the basement membrane zone that showed unusual serrated patterns or did not display a predominant intensity for C3, it was not possible to distinguish between bullous pemphigoid and epidermolysis bullosa acquisita because reliable differentiation between these two disease entities requires identifying the exact location of the immune complex’s deposition and the level of the bulla split by immunological tissue-based assays or by detecting specific circulating antibodies in serology [24,25]. It is beyond the scope of this study to break down the serrated patterns of DIF.

In the intraepidermal blister subgroup, the observed agreement between the clinical and histological diagnoses was high (81.8% in Table 4). However, among the nine cases with disagreements between the clinical and histological diagnoses, the histological diagnosis was in concordance with the final diagnosis in eight out of nine cases, while the clinical diagnosis was in concordance with the final diagnosis in only one out of nine cases. The difference between the clinical and histological diagnoses was statistically significant (mid-*p*-McNemar’s test, mid-*p*-value = 0.021). These findings indicate that histological diagnoses have a higher degree of accuracy than clinical diagnoses. Similarly, DIF diagnosis was concordant with the final diagnosis in seven out of nine cases (Table 4), with a statistically significant difference between the clinical and DIF diagnoses (mid-*p*-value = 0.008). These findings indicate that DIF diagnoses have a higher degree of accuracy than clinical diagnoses.

Our study showed that within the intraepidermal blister subgroup, the histological diagnosis was in concordance with the final diagnosis in 96.4% of the cases. The histological diagnosis was based primarily on the plane of split formation and acantholysis. Acantholysis could also be seen in non-autoimmune bullous disorders, such as Hailey-Hailey disease, Darier disease, and Grover’s disease [1,2,3,26]. Published studies have shown that H and E diagnoses are 80% sensitive and 97% specific for intraepidermal blistering disorders, and it is especially accurate in the diagnosis of pemphigus vulgaris [27]. We demonstrated that the histological and DIF diagnoses were comparable in diagnosing intraepidermal blister disorders (mid-*p*-McNemar’s test, mid-*p*-value = 1.000). Moreover, we did not identify any cases in which both the histological and DIF findings were discordant with the final diagnosis. In summary, the utilization of both histological and DIF studies provides the necessary information to derive the final diagnosis in all cases of the intraepidermal blister subgroup in the study.

In the subepidermal blister subgroup, the in-pair difference of the three diagnostic methods was not statistically significant according to mid-*p*-McNemar’s test (*p* > 0.05). However, the histological diagnosis was concordant with the final diagnosis in only 64.9% of the subepidermal blister cases, whereas concordance between the clinical diagnosis and final diagnosis was 81.1%, and between DIF diagnosis and the final diagnosis was 83.8%. We based the histological diagnosis of subepidermal disorders on two major findings: the presence of subepidermal blisters and the type of infiltrating inflammatory cells, which may vary according to the age of the lesion. The histological assessment appeared to have lower accuracy in evaluating subepidermal bullous disorders. A possible cause of the discordance is that a number of non-immunobullous disorders, such as drug allergy, hypersensitivity reactions, insect bites, herpetic infection, allergic contact dermatitis, and infectious skin bullae, are relatively common in Vietnam, and they have clinical features that may overlap with subepidermal bullous disorders and may have led to diverse and discordant clinical diagnostic assessments.

The Brennan-Prediger coefficient revealed a slight concordance (κ_BP_ = 0.17) between the clinical, histological, and DIF findings in the subepidermal group. Similarly, the concordance between the histological and clinical diagnoses, as well as between the histological and DIF diagnoses, was slight, with κ_BP_ values of 0.027 and 0.081, respectively. In contrast, there was better concordance between the clinical and DIF diagnoses (κ_BP_ = 0.41). These findings suggest that the limited diagnostic accuracy of routine H and E interpretations for classifying subepidermal bullous disorders could influence the overall degree of concordance between the clinical, histological, and DIF diagnoses in this subgroup.

A limitation of our study is that we often lacked comprehensive details of the clinical evaluation of ABD, especially for cases that originated from general practitioners, internists, and other non-dermatologic clinicians. We gathered clinical diagnoses from patients’ medical reports to consider as a reference for formulating the final diagnoses. Without detailed information on the clinical criteria which clinicians utilized for their clinical diagnoses, we were unable to conclude accurate correlations between the clinical diagnosis and histopathologic diagnosis.

Other immunological methodologies, such as serologies, ELISA, and indirect immunofluorescent staining with the salt-split skin technique, could improve the diagnostic accuracy of immunobullous disorders. However, many patients in our study did not have access to these assays other than standard H and E, and DIF for diagnostic work-ups. Within the scope of the clinical, histological, and DIF findings in this study, we utilized the classic histological and DIF characteristics of each subtype of bullous disorders to arrive at the final diagnosis. This also reflects the routine and practical approach we use in our daily medical practice in diagnosing bullous disorders in Vietnam. In summary, our study demonstrates that the diagnosis of autoimmune bullous disorders in the Vietnamese population could be accurately formulated based on a combination of clinical, histological, and DIF studies.

## 5. Conclusions

The assessment of autoimmune bullous dermatoses in Vietnamese patients shows that the clinical, histological, and DIF evaluations had a moderate level of agreement in intraepidermal bullous disorders, while these three diagnostic modalities had a slight level of agreement in subepidermal bullous disorders. The histological and DIF evaluations showed strong concordance in the intraepidermal group, but it appeared to have a limited role in the subepidermal group. In cases with typical clinical and histological features, DIF stains confirmed the diagnosis. However, in cases in which the clinical features and histopathologies were inconclusive, the final diagnosis was concluded based on additional DIF studies. In real-world diagnostic evaluations, a combination of clinical, histological, and DIF studies would provide an optimal diagnostic work-up.

## Figures and Tables

**Table 1:** Criteria For the Histological Diagnosis of Autoimmune Bullous Dermatoses.

Histological Criteria	Histological Diagnoses
Suprabasilar acantholysis of the epidermis and/or the follicular epithelium	Deep pemphigus (PV and PNP)
Subcorneal or intragranular acantholysis of the epidermis and/or the follicular epithelium	Superficial pemphigus (PF, PE, PH, and the subtype of IAP)
Intraepidermal blister with an uncertain level of splitting and/or without acantholysis	Unclassified intraepidermal blister
Subepidermal, unilocular blister with eosinophilic infiltration	BP
Subepidermal, multilocular blister with predominantly neutrophilic infiltration	LAD/BSLE/MMP/anti-p200 pemphigoid/inflammatory EBA
Subepidermal blister with predominantly neutrophilic infiltration and microabscesses of the dermal papillae	DH
Subepidermal without histological characteristics of BP, LAD, and DH	Unclassified subepidermal blister

BP = bullous pemphigoid, BSLE = bullous systemic lupus erythematosus, DH = dermatitis herpetiformis, EBA = epidermolysis bullosa acquisita, IAP = immunoglobulin A pemphigus, LAD = linear IgA dermatosis, MMP = mucous membrane pemphigoid, PE = pemphigus erythematosus, PF = pemphigus foliaceous, PH = pemphigus herpetiformis, PNP = paraneoplastic pemphigus, PV = pemphigus vulgaris.

**Table 2:** Criteria For the Direct Immunofluorescence Stain Diagnosis of Autoimmune Bullous Dermatoses.

Type of Immunoglobulin	Site of Immunoglobulin Deposition	Pattern of Immunoglobulin Deposition	Intensity of Immunoglobulin Deposition	DIF Diagnosis
IgG +/− C3	Epidermal cells; no BMZ staining	Linear/fine granular	Throughout the entire epidermis or more intense in the lower layer	PV
IgG +/− C3	Epidermal cells; no BMZ staining	Linear/fine granular	Throughout the entire epidermis or more intense in the upper layer	PF
IgG +/− C3	Epidermal cells and BMZ	Linear/fine granular	-	PNP/PE
IgA	Epidermal cells	Linear/fine granular	-	IAP
IgA	Dermal papillae	Fine granular	-	DH
IgG + C3	BMZ	N-shaped linear pattern	C3 > IgG	BP/PG
IgG with/without other types of immunoglobulins	BMZ	U-shaped linear pattern	IgG > C3	EBA/BSLE
IgA	BMZ	Linear	Mainly IgA	LAD

BMZ = basement membrane zone, BP = bullous pemphigoid, BSLE = bullous systemic lupus erythematosus, DH = dermatitis herpetiformis, EBA = epidermolysis bullosa acquisita, IAP = Immunoglobulin A pemphigus, LAD = linear IgA dermatosis, PE = pemphigus erythematosus, PF = pemphigus foliaceous, PG = pemphigoid gestationis, PNP = paraneoplastic pemphigus, PV = pemphigus vulgaris.

**Table 3:** Patient Characteristics And Prevalence Of Autoimmune Bullous Dermatoses.

Group	Disease	Number of Cases (%) (Total Cases = 92)	Age Range (Years)	Median Age (Years)	Female-to-Male Ratio
**Intraepidermal group**	PV	41 (44.6%)	11–77	52	2.7:1
	PF	13 (14.1%)	22–82	48	2.2:1
	IAP	1 (1.1%)	11	-	1 Male
**Subepidermal group**	BP	27 (29.4%)	10–95	65	0.9:1
	LAD	8 (8.7%)	14–72	33.5	1:1
	BSLE	1 (1.1%)	16	-	1 Female
	DH	1 (1.1%)	54	-	1 Male

BP = bullous pemphigoid, BSLE = bullous systemic lupus erythematosus, DH = dermatitis herpetiformis, IAP = immunoglobulin A pemphigus, LAD = linear IgA dermatosis, PF = pemphigus foliaceous, PV = pemphigus vulgaris.

**Table 4:** Contingency Table For McNemar’s Test Comparing the Diagnostic Accuracy Between the Paired Diagnostic Methods Of the Intraepidermal And Subepidermal Blister Subgroups.

**Intraepidermal Blister Subgroup** **(*N* = 55)**	**Histological Diagnosis**	**Clinical Diagnosis (*N* and %)**		**Total**
		(+)	(−)	
	(+)	45 (81.8%)	8 (14.6%)	53 (96.4%)
	(−)	1 (1.8%)	1 (1.8%)	2 (3.6%)
	TOTAL	46 (83.6%)	9 (16.4%)	55 (100%)
	**DIF Diagnosis**	**Clinical Diagnosis (*N* and %)**		**Total**
		(+)	(−)	
	(+)	46 (83.6%)	7 (12.8%)	53 (96.4%)
	(−)	0 (0%)	2 (3.6%)	2 (3.6%)
	TOTAL	46 (83.6%)	9 (16.4%)	55 (100%)
	**DIF Diagnosis**	**Histological Diagnosis (*N* and %)**		**Total**
		(+)	(−)	
	(+)	51 (92.8%)	2 (3.6%)	53 (96.4%)
	(−)	2 (3.6%)	0 (0%)	2 (3.6%)
	TOTAL	53 (96.4%)	2 (3.6%)	55 (100%)
**Subepidermal Blister Subgroup** **(*N* = 37)**	**Histological Diagnosis**	**Clinical Diagnosis (*N* and %)**		**Total**
		(+)	(−)	
	(+)	18 (48.6%)	6 (16.3%)	24 (64.9%)
	(−)	12 (32.4%)	1 (2.7%)	13 (35.1%)
	TOTAL	30 (81.1%)	7 (18.9%)	37 (100%)
	**DIF Diagnosis**	**Clinical Diagnosis (*N* and %)**		Total
		(+)	(−)	
	(+)	25 (67.6%)	6 (16.2%)	31 (83.8%)
	(−)	5 (13.5%)	1 (2.7%)	6 (16.2%)
	Total	30 (81.1%)	7 (18.9%)	37 (100%)
	**DIF Diagnosis**	**Histological Diagnosis (*N* and %)**		**Total**
		(+)	(−)	
	(+)	19 (51.4%)	12 (32.4%)	31 (83.8%)
	(−)	5 (13.5%)	1 (2.7%)	6 (16.2%)
	Total	24 (64.8%)	13 (35.2%)	37 (100%)

DIF = direct immunofluorescence stains. A result is designated as positive (+) if it is concordant with the final diagnosis. A result is designated as negative (−) if it is discordant with the final diagnosis.

**Table 5:** Differences Between the Paired Diagnostic Methods Of Autoimmune Bullous Dermatoses.

Pair of Diagnostic Methods	Number of Cases with Matching Diagnoses (Total *N* = 92)	Percentage of Matching Diagnoses	Mid-*p*-Value (Mid-*p*-McNemar’s Test, α = 0.05)
Clinical/Histology	63	68.5%	0.851
Clinical/DIF	71	77.2%	0.064
Histology/DIF	70	76.1%	0.134

DIF = direct immunofluorescence stains.

**Table 6:** Differences Between the Paired Diagnostic Methods Of the Intraepidermal Blister Subgroup.

Pair of Diagnostic Methods	Number of Cases with Matching Diagnoses (Total *N* = 55)	Percentage of Matching Diagnoses	Mid-*p*-Value (Mid-*p*-McNemar’s Test, α = 0.05)
Clinical/Histology	45	81.8%	0.021
Clinical/DIF	46	83.6%	0.008
Histology/DIF	51	92.7%	1.000

DIF = direct immunofluorescence stains.

**Table 7:** Differences Between the Paired Diagnostic Methods Of the Subepidermal Blister Subgroup.

Pair of Diagnostic Methods	Number of Cases with Matching Diagnoses (Total *N* = 37)	Percentage of Matching Diagnoses	Mid-*p*-Value (Mid-*p*-McNemar’s Test, α = 0.05)
Clinical/Histology	18	48.7%	0.167
Clinical/DIF	25	67.6%	0.774
Histology/DIF	19	51.4%	0.096

DIF = direct immunofluorescence stains.

**Table 8:** Discordant Findings Between the Clinical And Direct Immunofluorescence Stains For Subepidermal Blisters.

Case No.	Clinical Diagnosis	Histological Diagnosis	DIF Diagnosis	Final Diagnosis
58	Linear IgA dermatosis	Bullous pemphigoid (BP)	BP	BP
64	Herpes-associated erythema multiforme	BP	BP	BP
71	Linear IgA dermatosis	Unclassified pemphigoid	BP	BP
72	Dermatitis herpetiformis	BP	BP	BP
73	Linear IgA dermatosis	BP	BP	BP
79	Pemphigus vulgaris	BP	BP	BP

## Data Availability

Not applicable.
